# A trio of infectious diseases and pulmonary embolism: A developing world’s reality

**DOI:** 10.4102/sajhivmed.v22i1.1192

**Published:** 2021-01-28

**Authors:** Somasundram Pillay, Nombulelo Magula

**Affiliations:** 1 Department of Internal medicine, Faculty of Health Sciences, University of KwaZulu-Natal, Durban, South Africa

**Keywords:** HIV, tuberculosis, COVID-19, pulmonary embolism, middle-income countries

## Abstract

**Introduction:**

Human immunodeficiency virus (HIV), Tuberculosis (TB) and coronavirus disease (COVID-19) infections independently possess the ability to trigger formation of venous thromboembolism (VTE) and pulmonary embolism (PE). To the authors’ knowledge, this is the first case report describing the presence of PE in a patient with all three aforementioned infectious co-morbidities.

**Presentation:**

A patient living with HIV with virological failure secondary to defaulting antiretroviral therapy (ART) presented with hypoxia, clinical and radiological features suggestive of community-acquired pneumonia (CAP) with raised inflammatory markers and D-dimer levels.

**Management:**

She was commenced on prophylactic anticoagulation, supplemental oxygen and empirical antibiotics targeting CAP and pneumocystis jiroveci pneumonia, swabbed for COVID-19 infection and had sputa sent for Gene Xpert® TB testing. A day later, COVID-19 results returned positive and the patient was transferred to isolation and added onto dexamethasone and therapeutic anticoagulation. Sputa returned positive for mycobacterium TB a day later, and anti-tuberculosis therapy was added. She remained persistently hypoxic, with a Well’s score of 3 placing her at moderate risk for PE, which prompted for a computed tomography pulmonary angiogram (CTPA) being ordered, which demonstrated left lower lobe subsegmental PE. Warfarin was added to her regimen. She was discharged on day 18 with a therapeutic international normalised ratio (INR) and not requiring oxygen therapy.

**Conclusion:**

This scenario is relevant in low to middle-income countries. The utilisation of a raised D-Dimer in the setting of all four coexisting conditions in arriving at a definite diagnosis remains uncertain. We noted that despite our index patient being on thrombo-prophylaxis, she developed PE highlighting the need for increased vigilance in all COVID-19 patients, even those on prophylactic anticoagulation.

## Introduction

Coronavirus disease (COVID-19) infection is associated with increased prevalence of venous thromboembolism and pulmonary embolism (PE).^1,2,3,4,5^ Pulmonary embolism carries a high morbidity and mortality burden, and a high index of suspicion for underlying PE must be maintained in all COVID-19 infected patients. Hypercoagulability in COVID-19 infection occurs either because of altered haemostasis, severe inflammation, endothelial dysregulation or disseminated intravascular coagulation.^1^

The risk of venous thromboembolism (VTE) in patients living with HIV (PLHIV) is increased in patients who are antiretroviral therapy (ART)-naïve, those with a low cluster of differentiation (CD4) counts and virally unsuppressed.^6^

Dentan and colleagues showed that tuberculosis is significantly associated with VTE. They, however, found no link between TB and PE but postulated that the occurrence of PE in patients with TB (PWTB) could be explained by hypercoagulability.^7^ Reports of PE in PWTB in Africa are scarce. Kwas et al. have described three cases of TB associated with PE in patients from Tunisia,^8^ whilst Ekukwe and colleagues described another case of bilateral PE in a PWTB.^9^

Clinical and therapeutic challenges exist in PLHIV presenting with community-acquired pneumonia (CAP). Depending on the patient’s ART compliance, viral load, certain differentials arise – especially if the patient is viral unsuppressed where in addition to COVID-19 pneumonia, one considers pneumocystis jiroveci pneumonia (PJP) and/or pulmonary TB as differentials. Empirical therapy is commenced with an antibiotic (containing a combination of trimethoprim and sulfamethoxazole) plus corticosteroid therapy for PJP with amoxicillin-clavulanic acid and azithromycin for CAP.^10^

We describe the following typical patient encountered in clinical practice to demonstrate the need for maintaining a high level of clinical vigilance for PE in patients with coexistent HIV, TB and COVID-19 infection. No data exists, describing the occurrence of PE in such a patient co-infected with all three infectious conditions.

## Case report

**FIGURE 1:**
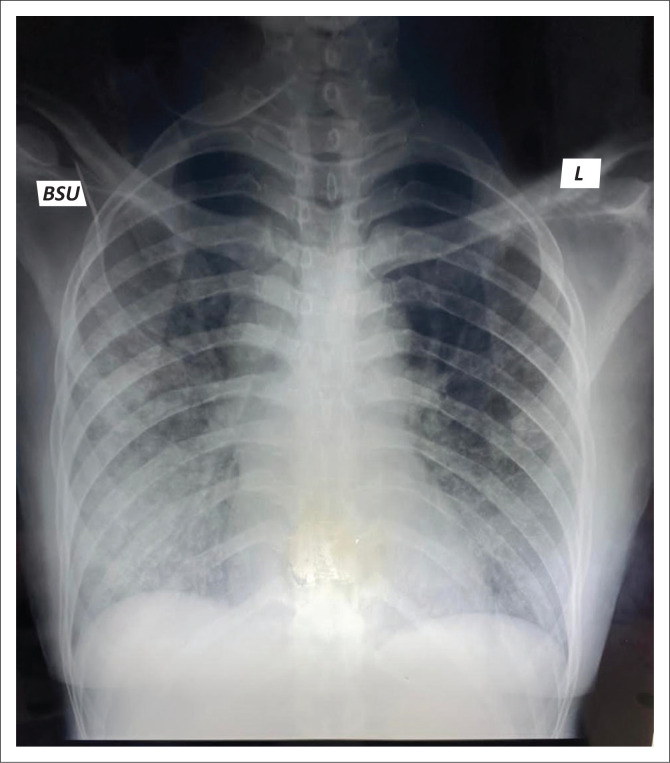
Chest radiograph (postero-anterior view).

Miss B.N.S., a 44-year-old female living with HIV since 1997, had defaulted ART since 2012 and was virologically unsuppressed (viral load of 23 358 copies/mL, CD4 = 66 cells/µL). She presented on 22/09/2020 with a 1-week history of constitutional symptoms (poor appetite, night sweats and easy fatigability) associated with a non-productive cough and shortness of breath. No symptoms of fever, sore throat, anosmia or dysgeusia were elicited. Clinical examination revealed a chronically ill-looking patient, tachycardia (133 beats/min), tachypnoea (22 breaths/min) and hypoxic at room air (oxygen saturation of 88%). Chest auscultation revealed crepitations in the mid and lower zones of her right lung. Chest radiograph (Figure 1) showed bilateral central and peripheral ground-glass opacification (GGO), greater involvement of the right lung.

Blood investigations revealed results as per Table 1. Based on the patient’s history, clinical, radiological and biochemical findings, a diagnosis of CAP was made, and she was started empirically on amoxicillin-clavulanic acid, azithromycin, oral trimethoprim/sulfamethoxazole, prednisone and prophylactic clexane® 60 mg daily. Nasopharyngeal swab and sputa were sent for COVID-19 and TB polymerase chain reaction (PCR). A day later, the COVID-19 PCR result returned as positive. She was initiated onto dexamethasone, vitamin D, zinc, thiamine, ascorbic acid and therapeutic anticoagulation (enoxaparin 60 mg BD). Whilst in the ward she remained hypoxic requiring 100% rebreather mask to maintain an oxygen saturation of over 90%. The sputa TB PCR test returned positive on 24/09/2020, and the patient was initiated onto anti-tuberculosis treatment (Rifafour® 3 tablets with pyridoxine 25 mg daily).

**TABLE 1:** Results of laboratory investigations.

Variables	22/09/2020	30/09/2020	04/10/2020	07/10/2020
C-reactive protein (CRP) mg/L [range < 10 mg/L]	90	182	83	-
White blood cell count [range * 10^9^/L]	11.9	11.0	6.7	-
Procalcitonin [range 0.25 ug/L – 0.5 ug/L indicating possibility of localised bacterial infection]	-	0.35	-	-
D-Dimer [Range 0.0 mg/L – 0.25 mg/L]	-	1.10	-	-
International normalised ratio (INR) [range 0.89–1.13]	-	1.26	3.40	3.92
Beta-D-glucan [Positive > 80 pg/mL]	-	> 500	-	-
Hepatitis A, B, C serology	Negative	-	-	-
Rapid plasma reagin (RPR)	Negative	-	-	-
Cryptococcal antigen test	Negative	-	-	-

**FIGURE 2:**
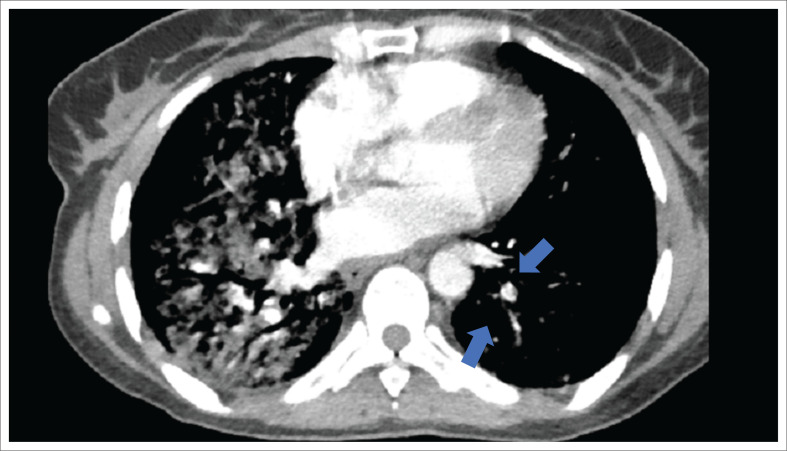
Computed tomography pulmonary angiogram showing filling defect in left lower lobe subsegmental pulmonary artery.

However, despite being on treatment for PJP, pulmonary TB and CAP, the patient remained hypoxic with a Wells’ score of 3 (heart rate > 100 bpm and prolonged immobilisation) putting her at moderate risk of developing a PE.^11^ A CTPA was ordered and revealed bilateral GGO and left lower lobe subsegmental pulmonary emboli (Figure 2).

No evidence of deep vein thrombosis was found. Warfarin 5 mg daily was added to her existing anticoagulation regimen.

The final diagnosis made in Miss B.N.S. was that of a PLHIV, virologically unsuppressed, who defaulted ART with a CD4 of 66 with confirmed:

- Severe COVID-19:^12,13,14^
- Pulmonary tuberculosis
- Pulmonary embolism
- *Pneumocystis iirovecii* pneumonia (clinical diagnosis plus a positive serum B-D-glucan).

She remained hypoxic needing prolonged oxygen supplementation until Day 17 post-admission. On Day 18, she was transferred to a step-down facility off oxygen to continue with her anticoagulation and TB treatment with a view to restarting ART in 6–8 weeks.

## Discussion

Individually, HIV, TB and COVID-19 can predispose to thromboembolism. This case report describes the presence of PE in a patient with all aforementioned infectious diseases and the complexities associated with diagnosis in a developing country. No literature could be found on the combination of HIV, TB, COVID-19 and the development of PE in a single patient.

Daily clinical patient re-evaluation is a necessity even in the times of COVID-19. This is often neglected as clinical staff are afraid of their well-being and resort to making notes without careful patient re-examination. Together with clinical examination, review of blood investigations and patient’s oxygen consumption needs, a management plan could be formulated.

The plasma D-dimer test has a low specificity and can be raised in a multitude of conditions including HIV-infection, TB, VTE, PE, COVID-19, pneumonia and increased age.^15,16,17,18^ This limited its use in differentiating between the four coexistent medical conditions in our patient, all of which are known to cause elevations in D-dimer levels. This highlights the complexity of diagnosing PE in the context of HIV, TB and COVID-19 infection.

The debate on the use of thromboprophylaxis in patients hospitalised with COVID-19 is ongoing with most studies currently advocating for its use.^19,20,21^ Prophylactic anticoagulation has long been recommended for at-risk in-patients with medical conditions such as HIV and/or TB.^22,23^ Despite being on prophylactic enoxaparin, our patient still developed a PE. This is similar to what Klok et al. and Tang et al. showed in their studies.^9,24^

Pulmonary embolism remains a great masquerader and clinicians must maintain a level of vigilance for diagnosing this life-threatening medical condition, more especially during the COVID-19 infection era.
